# What Is the Population Structure of Poecilogonic Species? Evidence From Large‐Scale Genotyping in a Neogastropod Lineage (Conoidea: *Raphitoma*)

**DOI:** 10.1111/mec.70170

**Published:** 2025-11-11

**Authors:** Giacomo Chiappa, Giulia Fassio, Maria Vittoria Modica, Nicolas Puillandre, Marco Oliverio

**Affiliations:** ^1^ Department of Biology and Biotechnologies “Charles Darwin” Sapienza University of Rome Rome Italy; ^2^ Department of Biology and Evolution of Marine Organisms Stazione Zoologica Anton Dohrn Rome Italy; ^3^ Institut Systématique Evolution Biodiversité (ISYEB), Muséum National D'histoire Naturelle, CNRS, Sorbonne Université, EPHE, Université Des Antilles Paris CP France

**Keywords:** dispersal, poecilogony, population genetic, *Raphitoma*, SLAF‐seq

## Abstract

Dispersal of marine benthic invertebrates is typically dependent on the developmental mode of their pelagic larvae, which can be prolonged and based on plankton feeding (planktotrophic), or short and rely on the nutrients from the egg (non‐planktotrophic). The uncommon poecilogonic species can commit to both developmental modes, with remarkable implications for their population genetics, not yet fully investigated. In this study, we obtained reduced‐representation genome‐wide SNP data for three species of the neogastropod genus *Raphitoma* Bellardi, 1847 from the Mediterranean, including the putatively poecilogonic species *Raphitoma philberti* (Michaud, 1829) and *R. laviae* (R. A. Philippi, 1844). A total of 80 samples were sequenced to test the poecilogony and analyse the population genetics of *R. laviae*. Although a certain degree of segregation was highlighted between the planktotrophic and non‐planktotrophic samples in both species, they were ultimately found within conspecific bounds, confirming poecilogony. A set of loci that split samples with different development was identified, suggesting that a genetic component may be involved in poecilogony in both species. The population genetics of *R. laviae* fit patterns of both long‐ and short‐dispersal species: in Corsica, with only planktotrophic developers, no geographic structure was detected, whereas in Croatia, with only non‐planktotrophic developers, the geographic structure was present among localities 15–40 km apart. Notably, the species delimitation based on genome‐wide SNP data was contrasting with the one assessed in past studies, reiterating that a solid taxonomy (still not achieved) is paramount to correctly interpret the evolution of larval development in this group.

## 1 Introduction

In marine benthic invertebrates, where adults have limited (if any) vagility, larvae are usually entitled to dispersal and are crucial for maintaining gene flow among populations (Cowen and Sponaugle 2009; Jablonski and Lutz 1983; Modica et al. 2017; Travis and Dytham 1998; Wang and Altermatt 2019; Wray 1995). A variety of reproductive strategies and larval developments have evolved to maximise dispersal, including planktotrophic larvae, which can travel far distances while feeding autonomously, and are usually produced in large numbers to cope with mortality during the pelagic larval phase. Among the advantages of a planktotrophic development is also the reduction of intraspecific competition. In contrast, low‐dispersal reproductive strategies sacrifice the dispersal potential to favour higher local or short‐distance recruitment; invertebrates specialised in this approach rely on lecithotrophic larvae, which are usually produced in lower amounts and get their sustenance from the egg. In benthic invertebrates, these contrasting reproductive strategies can affect the speciation mode and rates (Jablonski and Lutz 1983; Krug et al. 2015; Oliverio 1996).

Each species usually relies on a single strategy, with the rare exceptions of some polychaete annelids and sacoglossan gastropods (Krug 2007; Zakas and Wares 2012), where different larval developmental modes have been observed in the same species or population, a condition known as poecilogony (Giard 1905). Being able to choose between high‐ and low‐dispersal developmental strategies allows combining the advantages of local recruitment and of gene flow, with profound implications for the ecology of marine species and their developmental evolution (Chia et al. 1996; Zakas and Wares 2012).

In a strict definition (Hoagland and Robertson 1988; Bouchet 1989), poecilogony was adopted to encompass only the larval phenotypic variation among the offspring of a single individual. The use of a restricted definition helps avoid the traps of mixing cryptic species with distinct developmental modes. However, such a restricted definition may mask the actual variety of larval phenotypic variation, hampering a comparative approach that may be crucial for understanding the evolutionary dynamics of developmental modes (Knott and McHugh 2012). At the same time, not all developmental variations are conveniently included within poecilogony: for instance, we agree that polymorphism in dispersal without other phenotypic differences among larvae than variation in the time to hatching (e.g., Clemens‐Seely and Phillips 2011; Przeslawski 2011) should not be included in the definition of poecilogony (Krug 2009). Therefore, it is useful to return to something closer to the original Giard's (1905) broad definition of poecilogony, addressing for every case its geographic‐ecological scale (variation in the offspring of a single individual; offspring of different individuals from the same population; offspring of individuals from different populations; offspring of the same individual or individuals from the same population, but in different seasons or in response to different environmental conditions). The adoption of a broader definition is therefore the most suitable for a truly comparative approach. However, it should be a priority for any study on poecilogony to take into careful consideration the taxonomy of the group, and rule out potentially cryptic species (Knott and McHugh 2012).

Current studies on poecilogonic species aim at understanding the biological mechanisms behind poecilogony, as well as the evolutionary processes that favour a mixed reproductive strategy against a purely planktotrophic or lecithotrophic one (Krug 2009). Regrettably, only very few species are known to exhibit poecilogony, and finding new cases implies the difficult task of observing and studying larval development. Shelled gastropods are optimal model organisms as they retain their embryonic/larval shell (protoconch) as adults, and the modality of the pelagic phase can be presumed directly from protoconch length and sculpture (Jablonski and Lutz 1983; Shuto 1974). The larval shell is, in fact, multi‐spiral in planktotrophic larvae and paucispiral in non‐planktotrophic larvae. Based on the presence of very similar or identical adult shells but different protoconchs, poecilogony has been suggested in several species of Gastropoda. However, almost all cases of poecilogony in non‐sacoglossan gastropods have been confidently dismissed, recognising in each case either distinct taxa with different development, erroneous assignment of larvae to adults, or poecilogony assumed only on the egg morphology (Bouchet 1989; Fassio et al. 2022; Hoagland and Robertson 1988). Consequently, the type of protoconchs in shelled gastropods (and thus their developmental strategy) is still considered a species‐level diagnostic character (Bouchet 1989; Oliverio 1996).

Nevertheless, a convincing poecilogonic species was subsequently discovered in the family Calyptraeidae (McDonald et al. 2014), and candidate cases were highlighted more recently in the neogastropod family Raphitomidae, the most diverse taxon of Conoidea (Bouchet et al. 2011; Kantor and Taylor 2002). Species delimitation within the genus *Raphitoma* was performed to test the taxonomic status of sibling species differing in larval development (Russini et al. 2020). Two species—preliminarily identified as *R. philberti* (Michaud, 1829) and *R. laviae* (R. A. Philippi, 1844)—included specimens exhibiting both multi‐spiral and paucispiral protoconch, revealing two candidate cases of poecilogony. In a subsequent study, a third species (namely “*Raphitoma* sp. C”), composed of planktotrophic specimens only, was documented as a member of this species complex (Chiappa et al. 2025). The assessment by Russini et al. (2020) was based on a Sanger dataset of mitochondrial (*cox‐1*, *16S rDNA*, *12S rDNA*) and nuclear (*ITS2*) markers. Although the claim of poecilogony seems well supported by this data, it is also argued that the analyses may have failed to split the multi‐spiral and paucispiral samples within *R. philberti* and *R. laviae* because of a lack of resolution of the dataset (Russini et al. 2020).

Confirming poecilogony is of great importance from a taxonomic perspective, as it implies that larval development, and therefore protoconch morphology, may vary in some species (potentially leading to incorrect species delimitations and biased connectivity assessments: see Pante et al. 2015), and from an evolutionary perspective, as poecilogony could be the intermediate condition leading to speciation by loss of planktotrophy in at least one of the descending lineages (Oliverio 1996). Furthermore, the few sacoglossan species known so far as poecilogonic among gastropods are all herbivores, and it has been hypothesised that poecilogony could be an adaptation to trophic source fluctuations (Krug 2009; Strathmann 1978b, 1978a). Nevertheless, raphitomids are predators (Chiappa et al. 2024), which would represent a novel, unknown ecological combination.

Using the candidate cases of poecilogony identified by Russini et al. (2020), we performed a reduced‐representation sequencing (RRS) on samples of *R. philberti*, *R. laviae* and *Raphitoma* sp. C, to test the following hypotheses:

- That one or two poecilogonic species are identified in the genus *Raphitoma* using genome‐scale data, confirming the species delimitation pattern suggested by Sanger data (Russini et al. 2020), or, alternatively, that non‐poecilogonic, discrete units are identified in the species complex, thus rejecting the claims of poecilogony;
- Whether a poecilogonic species shows a global geographic pattern of genetic variation similar to that of a high‐dispersal species (due to the presence of planktotrophic larvae), or the genetic structure features typical of a low‐dispersal species (due to the presence of lecithotrophic larvae), or both.

## 2 Material and Methods

### 2.1 Dataset

The dataset included 80 specimens identified as *Raphitoma philberti*, *R*. sp. C and *R. laviae*, following genetic assessment (Chiappa et al. 2025; Fassio et al. 2019; Russini et al. 2020) sampled from three main regions in the Mediterranean (Corsica + Sardinia, Croatia and Greece). The type of larval development was assessed by observation of protoconch morphology: multi‐spiral (two or more whorls) with cancellated sculpture for planktotrophic development; paucispiral with finer, orthogonal sculpture for non‐planktotrophic development. For clarity, we will directly refer to specimens as planktotrophic or non‐planktotrophic hereafter, instead of referring to their protoconch morphology. The vouchers are stored in the Department of Biology and Biotechnologies “Charles Darwin”, Sapienza University of Rome (BAU), or in the Muséum National d'Histoire Naturelle, Paris (MNHN).

Samples were processed following a specific locus amplified fragment sequencing (SLAF‐Seq): DNA was double digested using enzymes RsaI and HaeIII, fragments were range selected, barcoded, amplified via PCR and sequenced in 150 PE on a NovaSeq machine (BMKGene—Münster, Germany). For additional details on the SLAF‐Seq protocol, see Sun et al. (2013). Following raw reads de‐multiplexing and quality trimming, the SLAF tags were clustered and polymorphic tags were identified. For each tag, reads were mapped on the highest depth sequence using BWA‐MEM v0.7.10 with ‐r 789 (Li and Durbin 2009) and SNP calling was performed by taking the output intersection of GATK v4.2.1.0 UnifiedGenotyper (McKenna et al. 2010) and samtools v1.9 mpileup (Li et al. 2009) commands, with default settings. The resulting dataset was filtered using vcftools v0.1.16 (Danecek et al. 2011), allowing a minimum allele frequency of 0.05, a minimum sequencing depth of 6× and only biallelic SNPs. Samples with more than 95% missing data were removed. Bioinformatic analyses requiring heavy computing resources were performed on Terastat2 (Bompiani et al. 2020). Two types of datasets were finally produced:

- A SNP dataset, including only allele information with less than 10% missing data. A second SNP dataset, including only a subset of samples with both larval development of *R. laviae*, was also assembled for population genetics (see results). A single random SNP per locus was selected for analyses requiring unlinked data.
- Three SLAF tag sets of ~100 high‐quality tags each: one with tags with less than 10% missing data in a subset of 32 samples representing all lineages while maximising completeness (dataset SLAF90); two randomly resampled from tags with less than 25% missing data shared by all samples (datasets SLAF75.1 and SLAF75.2).

### 2.2 Validation of Poecilogony

The SNP dataset was analysed to check if the species as identified previously with mitochondrial and nuclear markers (Chiappa et al. 2025; Russini et al. 2020) were consistently retrieved by a reduced genome dataset. Multiple approaches were adopted: a structure analysis performed with the R package LEA (Frichot and François 2015), estimating the number of ancestral populations (*K*) that minimises cross‐entropy (testing 1 to 10 *K*); a shared ancestry analysis computed using the R package fineSTRUCTURE (Lawson et al. 2012), ordering loci according to the Linkage Disequilibrium using the provided script for unmapped data; a Principal Component Analysis (PCA) performed on R with the prcomp command (R Core Team 2023) and plotted using the R package ggplot2 (Wickham 2016).

When a large number of loci is used, species delimitation methods are known to possibly “oversplit” (Sukumaran and Knowles 2017), as they may retrieve populations as distinct species regardless of gene flow (Leaché et al. 2019; Rannala and Yang 2020). In order to evaluate whether the populations identified from the SNP dataset analyses were conspecific, the SLAF tags datasets were analysed with a Bayesian approach using the species delimitation algorithm bpp 4.0 (Flouri et al. 2018; Yang 2015), by estimating the genealogical divergence index (*gdi*; Jackson et al. 2017), which gives the probability that samples in two groups can coalesce in a given time (Poelstra et al. 2021; Rannala and Yang 2020). Following Jorna et al. (2021), we performed multiple analyses on three SLAF tag subsets and took the consensus among them as the final results, allowing the retrieval of the most robust signal with approachable computational costs. Additional information on the calibration of this analysis is in Appendix S1‐1. We used Jackson's et al. (2017) rule of thumb, where *gdi* < 0.2 indicates a single species, *gdi* > 0.7 indicates two species and values in‐between represent an ambiguous grey zone. Nucleotide diversity (*π*) and divergence (*D*_*xy*_), and net divergence (*D*_*a*_) among the units retrieved from the structure analyses were also computed for each SLAF tag subset (Nei and Li 1979) using MEGA X (Kumar et al. 2018).

To retrieve loci possibly related to larval development, *F*_*ST*_ per site was computed between a subsample (see Results) of planktotrophic and non‐planktotrophic specimens of *R. laviae* using Weir and Cockerham's (1984) formula with vcftools v0.1.16 (Danecek et al. 2011). Negative estimates were set to 0. Loci including sites with scores above the 95% quantile were selected and filtered from the SNP dataset. PCAs were then performed again to visualise the effects of this set of loci on the total variance.

### 2.3 Divergence Rates and Time Estimation

To date the historical events within the group of interest, we performed a time‐calibrated ML phylogenetic reconstruction of Raphitomidae using a *cox‐1* + *16S rDNA* dataset comprising 28 species of eight genera, after Almón et al. (2024).

Four constrained age intervals based on fossil data were used in the Raphitomidae analysis: (1) the origin of the family is assumed to have occurred 47.8–37.8 million years ago (Mya) based on the first known representative, the Middle Eocene *Pleurotomella polycolpa* (Cossmann, 1889) (Gougerot and Le Renard 1981; Lozouet 2017), a dating confirmed by molecular assessment (Abdelkrim et al. 2018); (2) the age of the mrca of European raphitomids (including the lineages corresponding to the modern *Leufroyia, Raphitoma* and *Cyrillia*) can be dated at 28–23 Mya, based on Upper Oligocene Raphitomidae with morphology more similar to *Leufroyia* than to *Raphitoma* (e.g., *R. subfragilis* Lozouet 2017); (3) the age of the mrca of the *Raphitoma*‐like lineages (*Raphitoma* and *Cyrillia*) can be placed in the Middle Miocene (16.3–12.8 Mya) when species recalling modern forms – like the Badenian Paratethys *Raphitoma praehispida* (Boettger, 1906) – are first found; and (4) the split between the lineage leading to the Plio‐Pleistocene *Raphitoma histrix* and then to the Recent *R. pseudohystrix* (with its oldest representative, the Tortonian *Raphitoma dellabellaorum* Landau et al. 2020), and the rest of the NE Atlantic and Mediterranean *Raphitoma*, is dated at 11.6–7.2 Mya (Landau et al. 2020). Additional information on this analysis is in Appendix S1‐2.

### 2.4 Population Genetics of a Poecilogonic Species

A SNP dataset including a subset of planktotrophic and non‐planktotrophic samples in *R. laviae* (see Results) was analysed to explore the population genetics of a poecilogonic species, including a total of 47 specimens representing both larval developmental strategies. Instead of looking for the most likely number of populations in the dataset (e.g., the one with the lowest cross‐entropy), we preliminarily delimited populations according to the three main sampling regions: Corsica, Croatia and Greece. Then, we looked further at the micro geographic scale by partitioning the dataset according to the distribution of the sampling sites within Corsica (with samples from the Northern, Western and Southern coasts), and within Croatia (with samples from Punta Križa and the region spanning ~25 km around Ravni Kotari). Population genetics statistics were computed using the “populations” module of STACKS v2.76 (Catchen et al. 2011, 2013). A structure analysis was performed using LEA (Frichot and François 2015) setting the initial number of ancestral populations to three to test for geographic structure in the sampled areas. Isolation by Distance (Wright 1943) was tested using geodetic pairwise oceanographic distances measured on QGis v. 3.12.2 and a pairwise nucleotide distance matrix computed using vcf2dis (available at https://github.com/BGI‐shenzhen/VCF2Dis). Mantel tests (Mantel 1967) were performed with the Spearman model and 10,000 replicates with the R packages vegan v2.6.4 (Dixon 2003) and tidyverse v2.0.0 (Wickham et al. 2019).

## 3 Results

### 3.1 Dataset

The dataset composition (Table 1) included 13 specimens of *Raphitoma philberti* from Sardinia (*n* = 1), Corsica (*n* = 3), Greece (*n* = 2) and Croatia (*n* = 7), nine of *Raphitoma* sp. C. from Sardinia (*n* = 1), Corsica (*n* = 6) and Greece (*n* = 2), and 58 of *Raphitoma laviae* from Corsica (*n* = 35), Greece (*n* = 2) and Croatia (*n* = 21). The attribution of larval development by protoconch observation revealed 7 non‐planktotrophic specimens in *R. philberti*, including one from Corsica and 11 in *R. laviae*, all from Croatia.

**TABLE 1:** List of *Raphitoma* included in this study.

Sample ID	Larval develop	Region	Locality	Pop	Total reads	SLAF number	Miss rate	NCBI Biosample
*Raphitoma philberti*								
BAU 1888**	*non‐pl*	Italy	Taranto	—	43,580	4143	99.8	SAMN47597843
BAU 2241.2*	—	Croatia	Biograd	P	17,627,994	524,635	74.2	SAMN47597844
BAU 2248.2*	—	Croatia	Vrsi	P	33,829,538	546,128	76.1	SAMN47597845
BAU 2252.2	*non‐pl*	Croatia	Zaton	P	19,806,532	501,471	76.8	SAMN47597846
BAU 2261.2	*pl*	Croatia	Biograd	P	21,060,522	376,808	84.3	SAMN47597847
BAU 3046*	*pl*	Greece	Vai Is.	P	19,121,126	566,470	71.8	SAMN47597848
BAU 3352.1	*non‐pl*	Croatia	Punta Križa	P	8,678,402	402,672	79	SAMN47597849
BAU 3352.2**	*non‐pl*	Croatia	Punta Križa	—	921,222	27,967	99	SAMN47597850
BAU 3352.3**	*non‐pl*	Croatia	Punta Križa	—	7,319,556	104,765	95.1	SAMN47597851
BAU 4301	—	Italy	Cor‐N	P	20,657,512	513,510	77.5	SAMN47597852
MNHN‐IM‐2019‐6068*	*non‐pl*	Corsica	Cor‐N	P	23,640,440	528,642	76.4	SAMN47597853
MNHN‐IM‐2019‐17259*	*pl*	Corsica	Cor‐W	P	19,339,866	490,878	75.2	SAMN47597854
MNHN‐IM‐2019‐18330*	*pl*	Corsica	Cor‐W	P	17,869,208	511,477	75.5	SAMN47597855
*Raphitoma* sp. C								
BAU 3545.1	*pl*	Greece	Leros Is.	C	21,833,116	414,178	82.3	SAMN47597856
BAU 3545.2*	*pl*	Greece	Leros Is.	C	25,183,848	507,083	76.4	SAMN47597857
BAU 4300*	—	Sardinia	Cor‐S	C	30,721,574	480,241	81	SAMN47597858
MNHN‐IM‐2019‐13292*	*pl*	Corsica	Cor‐S	C	17,271,624	462,920	79.8	SAMN47597859
MNHN‐IM‐2019‐13944*	*pl*	Corsica	Cor‐S	C	22,629,494	514,508	78.4	SAMN47597860
MNHN‐IM‐2019‐14607*	*pl*	Corsica	Cor‐S	C	38,144,402	620,817	75	SAMN47597861
MNHN‐IM‐2019‐17885	*pl*	Corsica	Cor‐W	C	14,681,778	456,363	78.7	SAMN47597862
MNHN‐IM‐2019‐18329	*pl*	Corsica	Cor‐W	C	14,339,814	472,601	76.8	SAMN47597863
MNHN‐IM‐2019‐18507*	*pl*	Corsica	Cor‐W	C	13,864,880	467,896	75.8	SAMN47597864
*Raphitoma laviae*								
BAU 2238.1*	*non‐pl*	Croatia	Biograd	L3	17,862,178	703,998	64.5	SAMN47597865
BAU 2238.3*	*non‐pl*	Croatia	Biograd	L3	23,416,166	746,026	62.3	SAMN47597866
BAU 2241.1	*non‐pl*	Croatia	Biograd	L3	25,428,916	593,366	73.8	SAMN47597867
BAU 2243.1*	*pl*	Croatia	Sukošan	L1	17,823,974	666,224	63	SAMN47597868
BAU 2243.2*	*pl*	Croatia	Sukošan	L1	19,820,810	643,919	65.6	SAMN47597869
BAU 2243.3	*pl*	Croatia	Sukošan	L1	20,512,588	619,748	68.4	SAMN47597870
BAU 2246.2	*pl*	Croatia	Zaton	L1	20,789,562	627,163	67.8	SAMN47597871
BAU 2246.3	*pl*	Croatia	Zaton	L1	19,563,282	564,078	72.4	SAMN47597872
BAU 2246.4	*pl*	Croatia	Zaton	L1	11,130,408	549,984	69	SAMN47597873
BAU 2247	*non‐pl*	Croatia	Biograd	L3	15,149,460	637,950	68.2	SAMN47597874
BAU 2249.1*	*non‐pl*	Croatia	Sukošan	L3	12,490,178	570,818	70.2	SAMN47597875
BAU 2249.2	*non‐pl*	Croatia	Sukošan	L3	13,146,510	570,592	71.8	SAMN47597876
BAU 2249.3	*non‐pl*	Croatia	Sukošan	L3	16,383,468	477,206	76.5	SAMN47597877
BAU 2251.2	*pl*	Croatia	Turanj	L1	16,126,654	538,295	72.7	SAMN47597878
BAU 2255*	*non‐pl*	Croatia	Sabunike	L3	16,715,602	646,719	66.6	SAMN47597879
BAU 2267	*non‐pl*	Croatia	Sabunike	L3	13,918,112	557,429	71	SAMN47597880
BAU 2268.2	*non‐pl*	Croatia	Biograd	L3	12,450,926	573,088	72.6	SAMN47597881
BAU 2270.1	*pl*	Croatia	Biograd	L1	11,036,018	534,254	70.1	SAMN47597882
BAU 2270.2	*pl*	Croatia	Biograd	L1	11,598,640	526,275	71.5	SAMN47597883
BAU 2363.2	*non‐pl*	Croatia	Biograd	L3	25,574,130	590,484	73	SAMN47597884
BAU 3358.2	*pl*	Croatia	Punta Križa	L1	17,136,184	553,114	71.2	SAMN47597885
BAU 3550*	*pl*	Greece	Leros Is.	*	25,231,780	674,351	67.6	SAMN47597886
BAU 3551	*pl*	Greece	Leros Is.	L1	16,148,146	539,193	72.2	SAMN47597887
MNHN‐IM‐2019‐4014*	*pl*	Corsica	Cor‐N	L2	25,825,530	763,427	60.8	SAMN47597888
MNHN‐IM‐2019‐4020*	*pl*	Corsica	Cor‐N	L2	13,659,394	587,962	69	SAMN47597889
MNHN‐IM‐2019‐4066	—	Corsica	Cor‐N	L2	13,766,474	583,750	69.2	SAMN47597890
MNHN‐IM‐2019‐4068*	*pl*	Corsica	Cor‐N	L2	15,591,622	678,562	62.4	SAMN47597891
MNHN‐IM‐2019‐4087	*pl*	Corsica	Cor‐N	L2	21,972,282	644,356	67.2	SAMN47597892
MNHN‐IM‐2019‐4094*	*pl*	Corsica	Cor‐N	*	27,515,058	693,000	64.7	SAMN47597893
MNHN‐IM‐2019‐5300	*pl*	Corsica	Cor‐N	L1	14,162,368	572,206	70.1	SAMN47597894
MNHN‐IM‐2019‐5306	*pl*	Corsica	Cor‐N	L1	13,501,756	569,194	69.7	SAMN47597895
MNHN‐IM‐2019‐5342	*pl*	Corsica	Cor‐N	L2	14,987,102	651,221	66.2	SAMN47597896
MNHN‐IM‐2019‐6022	*pl*	Corsica	Cor‐N	L2	14,320,572	607,104	68.2	SAMN47597897
MNHN‐IM‐2019‐6024	*pl*	Corsica	Cor‐N	L2	15,467,482	644,948	65.6	SAMN47597898
MNHN‐IM‐2019‐6025*	*pl*	Corsica	Cor‐N	L1	20,655,040	645,116	66.7	SAMN47597899
MNHN‐IM‐2019‐6026	*pl*	Corsica	Cor‐N	L2	11,783,640	583,195	68.1	SAMN47597900
MNHN‐IM‐2019‐6034*	*pl*	Corsica	Cor‐N	L2	28,775,204	736,832	62.4	SAMN47597901
MNHN‐IM‐2019‐6035*	*pl*	Corsica	Cor‐N	L2	23,458,906	692,349	64.4	SAMN47597902
MNHN‐IM‐2019‐6067	*pl*	Corsica	Cor‐N	L2	13,916,090	607,161	69.1	SAMN47597903
MNHN‐IM‐2019‐6069	*pl*	Corsica	Cor‐N	L2	14,397,358	613,299	69.2	SAMN47597904
MNHN‐IM‐2019‐6090	*pl*	Corsica	Cor‐N	L2	14,914,300	635,732	67.2	SAMN47597905
MNHN‐IM‐2019‐12026	*pl*	Corsica	Cor‐S	L2	11,158,584	537,028	70.4	SAMN47597906
MNHN‐IM‐2019‐12932*	*pl*	Corsica	Cor‐S	L2	18,311,870	637,215	66.4	SAMN47597907
MNHN‐IM‐2019‐13703*	*pl*	Corsica	Cor‐S	L2	36,880,462	820,390	59.6	SAMN47597908
MNHN‐IM‐2019‐13959*	*pl*	Corsica	Cor‐S	L1	19,585,334	635,153	66.9	SAMN47597909
MNHN‐IM‐2019‐14537	*pl*	Corsica	Cor‐S	L2	18,516,596	684,876	64.6	SAMN47597910
MNHN‐IM‐2019‐14594	*pl*	Corsica	Cor‐S	L2	18,330,742	667,113	66.9	SAMN47597911
MNHN‐IM‐2019‐16533	*pl*	Corsica	Cor‐W	L2	18,990,116	672,372	65.8	SAMN47597912
MNHN‐IM‐2019‐16949*	*pl*	Corsica	Cor‐W	L1	19,386,880	615,702	67.7	SAMN47597913
MNHN‐IM‐2019‐17159	*pl*	Corsica	Cor‐W	L2	17,390,480	670,593	64.6	SAMN47597914
MNHN‐IM‐2019‐17246*	*pl*	Corsica	Cor‐W	L2	40,912,682	765,119	61.8	SAMN47597915
MNHN‐IM‐2019‐17406	*pl*	Corsica	Cor‐W	L2	19,034,446	679,430	65.5	SAMN47597916
MNHN‐IM‐2019‐17663*	*pl*	Corsica	Cor‐W	L2	14,375,434	628,467	66.5	SAMN47597917
MNHN‐IM‐2019‐17690	*pl*	Corsica	Cor‐W	L2	12,192,298	589,108	67.9	SAMN47597918
MNHN‐IM‐2019‐17888	*pl*	Corsica	Cor‐W	L2	17,024,372	655,702	66.4	SAMN47597919
MNHN‐IM‐2019‐17942	*pl*	Corsica	Cor‐W	L2	10,882,632	562,118	70.1	SAMN47597920
MNHN‐IM‐2019‐18144	*pl*	Corsica	Cor‐W	L2	14,249,610	632,210	66.8	SAMN47597921
MNHN‐IM‐2019‐18145	*pl*	Corsica	Cor‐W	L2	17,820,428	677,517	64.8	SAMN47597922

*Note:* Asterisks mark samples included in the SLAF90 reduced dataset (*) or removed from the analysis (**). “Pop” refers to the results of the structure analyses (see Figures 1 and 2). “Larval development” was inferred from protoconch morphology (*pl*: Planktotrophic; *non‐pl*: Non‐planktotrophic). “Miss rate” refers to the percentage of missing tags. For additional sampling information refer to Chiappa et al. (2024) Supporting Information.

A total of 1.57 billion reads were outputted after sequencing (19,165,278 reads per sample on average), and 287,633 polymorphic SLAF tags with x10.17 mean depth were retrieved in total, resulting in 1,329,954 total SNPs. Three samples of *R. philberti* with > 95% missing data were removed. The average miss rate of tags in the remaining samples was 70%, with values ranging from 60% to 84%. The SNP dataset comprised a total of 10,111 SNPs from 1789 loci. The high‐quality SLAF tags datasets comprised 119 tags with > 90% completeness in a subset of 32 samples (dataset SLAF90), and 202 tags with > 75% completeness shared by all samples (split in SLAF75.1 and SLAF75.2).

### 3.2 Validation of Poecilogony

**FIGURE 1:**
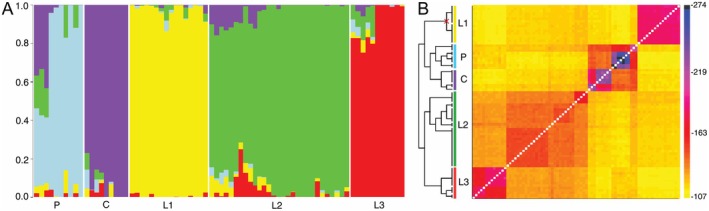
(A) Ancestry proportion of samples to five ancestral populations (*K*). (B) Matrix of co‐ancestry between samples. Colours correspond to ancestral populations retrieved in the structure analysis; all nodes had maximum support except the one crossed.

The optimal number of ancestral populations (*K*) resulting from the LEA analysis (Figure 1, additional plots provided in Figure S2) was five, corresponding to *Raphitoma* sp. C, *R. philberti* and three populations within *R. laviae*, hereafter called L1 (with samples from all localities, all planktotrophic), L2 (with samples from Corsica, all planktotrophic) and L3 (with samples from Croatia, all non‐planktotrophic). With *K* = 3, the analysis did not retrieve the three species as predetermined, but lumped *R. philberti* with *R*. sp. C and split L1 from the rest of *R. laviae*. Samples of population L1 were the first to split with *K* = 2. Three specimens of *R. philberti*, all planktotrophic, had mixed ancestry. In the fineSTRUCTURE analysis (Figure 1), high co‐ancestry values were estimated within the ancestral populations from the LEA analyses, and a geographical structure was present in non‐planktotrophic populations.

**FIGURE 2:**
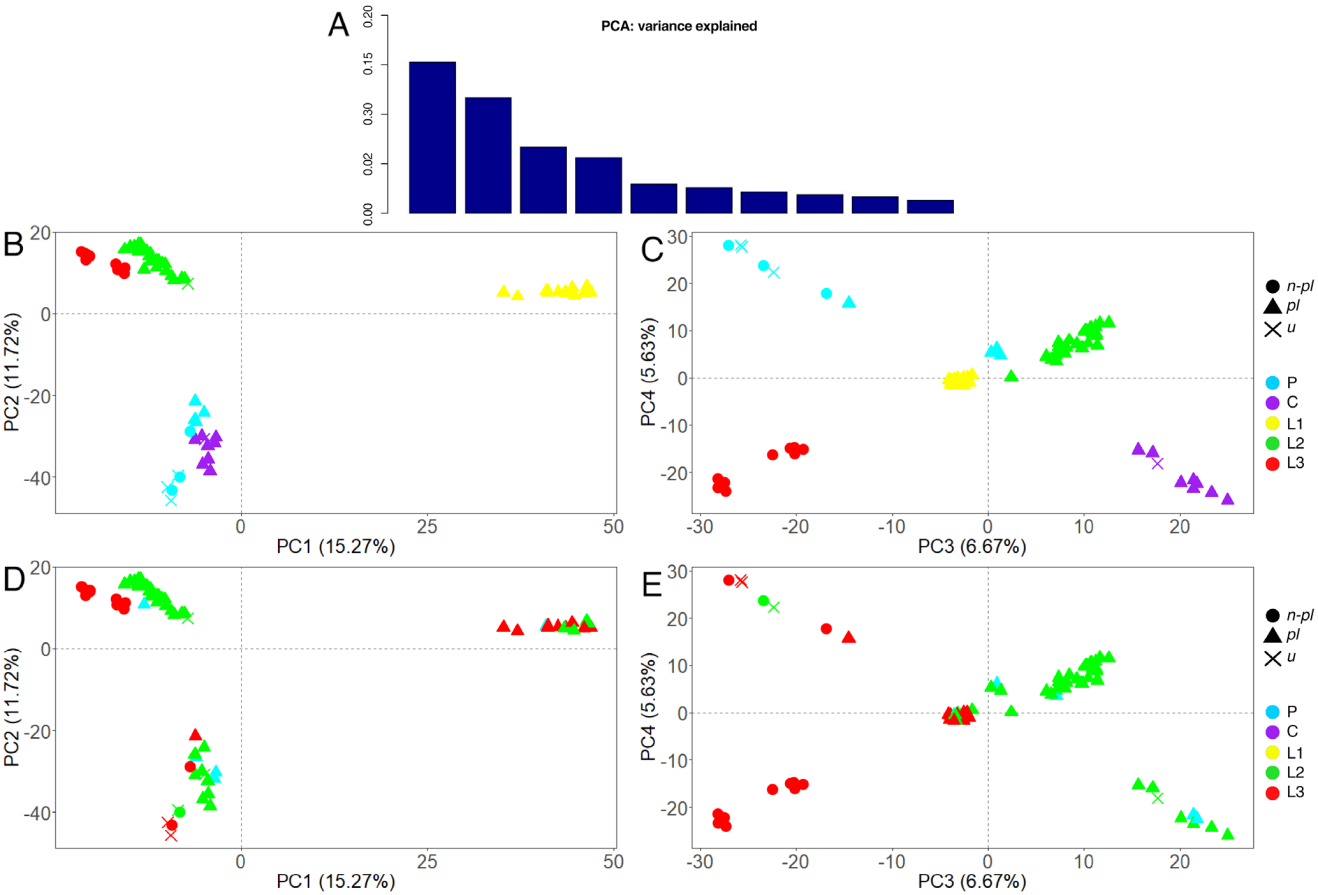
PCA plots with the first four principal components. (A) Proportion of variance explained by the first ten components. (B, C) Colour legend corresponding to groups retrieved in the structure analyses (Figure 1). (D, E) Colour legend corresponding to sampling localities (COR: Corsica; GR: Greece; KR: Croatia). Larval development inferred from protoconch morphology (*pl*: Planktotrophic; *non‐pl*: Non‐planktotrophic; *u*: Unknown).

The variance explained by the first two principal components of the PCA was 15.27% and 11.72%, rapidly decreasing to 6.67%, 5.63%, and below 3% in the following PCs (Figure 2, additional plots provided in Figure S3). Three main groups were identified for each species by plotting the samples onto the first two axes, not corresponding to the three species, but lumping *R. philberti* and *R. laviae* and separating L1 from the rest of *R. laviae*. Groups including planktotrophic specimens were very condensed, despite including samples from distant localities, whereas those that included non‐planktotrophic samples were more spread, often in accordance with their geographic distribution. The samples with different larval development were separated, with the exception of a single non‐planktotrophic specimen of *R. philberti* from Croatia (BAU 3352.1). In the plot resulting from the third and fourth PCs, the non‐planktotrophic samples were more evidently separated from the planktotrophic, with the planktotrophic specimens of *R. philberti* closer to the planktotrophic of *R. laviae* rather than the conspecific, non‐planktotrophic samples, with the only exception of a planktotrophic specimen of *R. philberti* from Croatia (BAU 2261.2). This plot also showed a broader spread of non‐planktotrophic samples compared to the others.

The estimation of the genealogical divergence index (*gdi*) on three independent high‐quality datasets (Table 2) showed values (0.08–0.11) consistently below the 0.2 threshold when merging the samples belonging to units L2 and L3, indicating that the two planktotrophic and non‐planktotrophic populations retrieved in *R. laviae*, although separated in the structure analysis and slightly apart in the PCA, belong to the same species. Including samples of the group L1, however, yielded values (0.19–0.26) not consistently below the threshold, thus not supporting conspecificity of L1 with L2 and L3. All remaining values, including those corresponding to the three preliminary species, ranged between 0.18 and 0.27. Nucleotide distance (*D*_*xy*_) among units ranged from 0.0052 to 0.0021 (Table 3). In particular, *D*_*xy*_ between L1 and the rest of *R. laviae* ranged from 0.0032 to 0.0041, whereas *D*_*xy*_ between L2 and L3 was lower, ranging from 0.0021 to 0.0027. Similarly, net divergence (*D*_*a*_) ranged from 0.0607 to 0.0004 (Table 3) and was higher between L1 and L2 or L3 (0.0028–0.0018) compared to the *D*_*a*_ between L2 and L3 (0.0009–0.0004).

**TABLE 2:** *Gdi* scores estimated from three SLAF tags datasets of 119 (SLAF90) and 100 (SLAF75) loci using three different priors for root age (*τ*) and genetic diversity (*θ*).

	*τ* = 5θ		*τ* = 0.5θ		*τ* = 0.05θ	
SLAF90						
L2L3	0.1088	0.1125	0.1082	0.1125	0.0993	0.1014
L1L2L3	0.2326	0.2120	0.2613	0.2369	0.2109	0.2065
PC	0.2383	0.2470	0.2390	0.2624	0.2382	0.2121
SLAF75.1						
L2L3	0.0852	0.081	0.078	0.0808	0.0934	0.0825
L1L2L3	0.2203	0.1967	0.1963	0.1896	0.2040	0.1944
PC	0.2328	0.2202	0.2309	0.2301	0.2284	0.2314
SLAF75.2						
L2L3	0.0971	0.0958	0.0957	0.1046	0.0954	0.0886
L1L2L3	0.2453	0.2348	0.2336	0.2421	0.2061	0.1951
PC	0.2731	0.2508	0.2711	0.2549	0.2443	0.2391

*Note:* Letters refer to the units as derived from the structure analysis (see Figure 1). Values represent the probability that samples in two groups can coalesce in a given time. Red scores are below the threshold for conspecific populations (*gdi* < 0.2).

**TABLE 3:** Genetic distance estimates from three SLAF datasets of 119 (SLAF90) and 100 (SLAF75) loci among units as derived from the structure analysis (see Figure 1). Left: Nucleotide diversity within (*π*) and divergence between (*D*_*xy*_) units. Right: Net divergence (*D*_*a*_) between units.

SLAF90	*π*	C	P	L1	L2	SLAF90	C	P	L1	L2
C	0.00269					P	0.00154			
P	0.00271	0.00424				L1	0.00302	0.00247		
L1	0.00099	0.00486	0.00432			L2	0.00188	0.00146	0.00186	
L2	0.00220	0.00433	0.00392	0.00346		L3	0.00238	0.00192	0.00213	0.00042
L3	0.00124	0.00435	0.00390	0.00324	0.00214					

SLAF75.1	*π*	C	P	L1	L2	SLAF75.1	C	P	L1	L2
C	0.00227					P	0.00164			
P	0.00207	0.00381				L1	0.00270	0.00235		
L1	0.00119	0.00443	0.00398			L2	0.00183	0.00176	0.00193	
L2	0.00214	0.00403	0.00386	0.00360		L3	0.00241	0.00218	0.00217	0.00061
L3	0.00134	0.00422	0.00388	0.00344	0.00235					

SLAF75.2	*π*	C	P	L1	L2	SLAF75.2	C	P	L1	L2
C	0.00268					P	0.00199			
P	0.00323	0.00495				L1	0.00320	0.00295		
L1	0.00132	0.00520	0.00522			L2	0.00234	0.00207	0.00205	
L2	0.00249	0.00492	0.00493	0.00395		L3	0.00304	0.00264	0.00278	0.00086
L3	0.00127	0.00501	0.00489	0.00407	0.00274					

**FIGURE 3:**
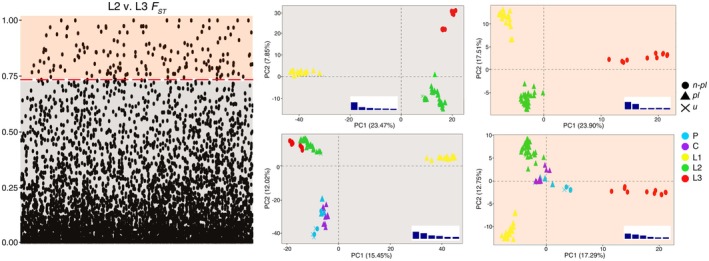
Left: *F*_*st*_ per site between planktotrophic (L2) and non‐planktotrophic (L3) samples in *R. laviae*. Dotted line represents the 95% percentile above mean. Right: PCA plots obtained with loci below and above the threshold, including only *R. laviae* and the complete dataset. Colour legend corresponding to groups retrieved in the structure analyses (Figure 1). Bar plots show the variance explained by the first six PCs.

A total of 114 loci were identified with sites showing *F*_*st*_ values above the 95% percentile threshold value (0.732) between the *R. laviae* planktotrophic samples in L2 and the non‐planktotrophic samples in L3. The PCA plots (Figure 3) obtained using loci below the threshold were very similar to those obtained with the all‐loci dataset. In *R. laviae*, one group of planktotrophic specimens (L2) was well separated from the other planktotrophic (L1), and was instead very close to a group of non‐planktotrophic samples (L3). In the PCA using only the set of loci above the *F*_*st*_ threshold (i.e., those with a divergence correlated with a difference in larval development in L2 v. L3), that same group became instead evidently closer to samples of the same development; furthermore, with these loci, the separation of non‐planktotrophic and planktotrophic specimens also involved *R. philberti*, which became fully split, without the exceptions retrieved using the full dataset.

### 3.3 Divergence Rates and Time Estimation

The likelihood‐ratio tests rejected the strict molecular clock hypothesis (*p* = 0.0000). For all major lineages, a clock‐like behaviour was also rejected on most nodes, suggesting the need for methods to estimate the age of nodes that allowed for different rates of molecular evolution among lineages. Cross‐validation analyses using a range of smoothing values from 0.1 to 10 yielded the best score for the lowest values. This indicated the need for a smoothing value that would allow for abrupt disparities in rates among close lineages. We thus used a value of 0.1 (however, we also used values up to 1, which produced similar results – data not shown).

Dating the Raphitomidae ML tree (Figure S1) yielded an age of 1.66 Mya (0.83–2.98) for the mrca of the three clades corresponding to *Raphitoma philberti, R*. sp. C and *R. laviae*. The mrca of the sibling species *R. cordieri* (Payraudeau, 1826) and *R. horrida* (Monterosato, 1884)—a planktotrophic and a non‐planktotrophic species, respectively—was dated at 3.3 Mya (1.81–8.81).

### 3.4 Population Genetics of a Poecilogonic Species

**FIGURE 4:**
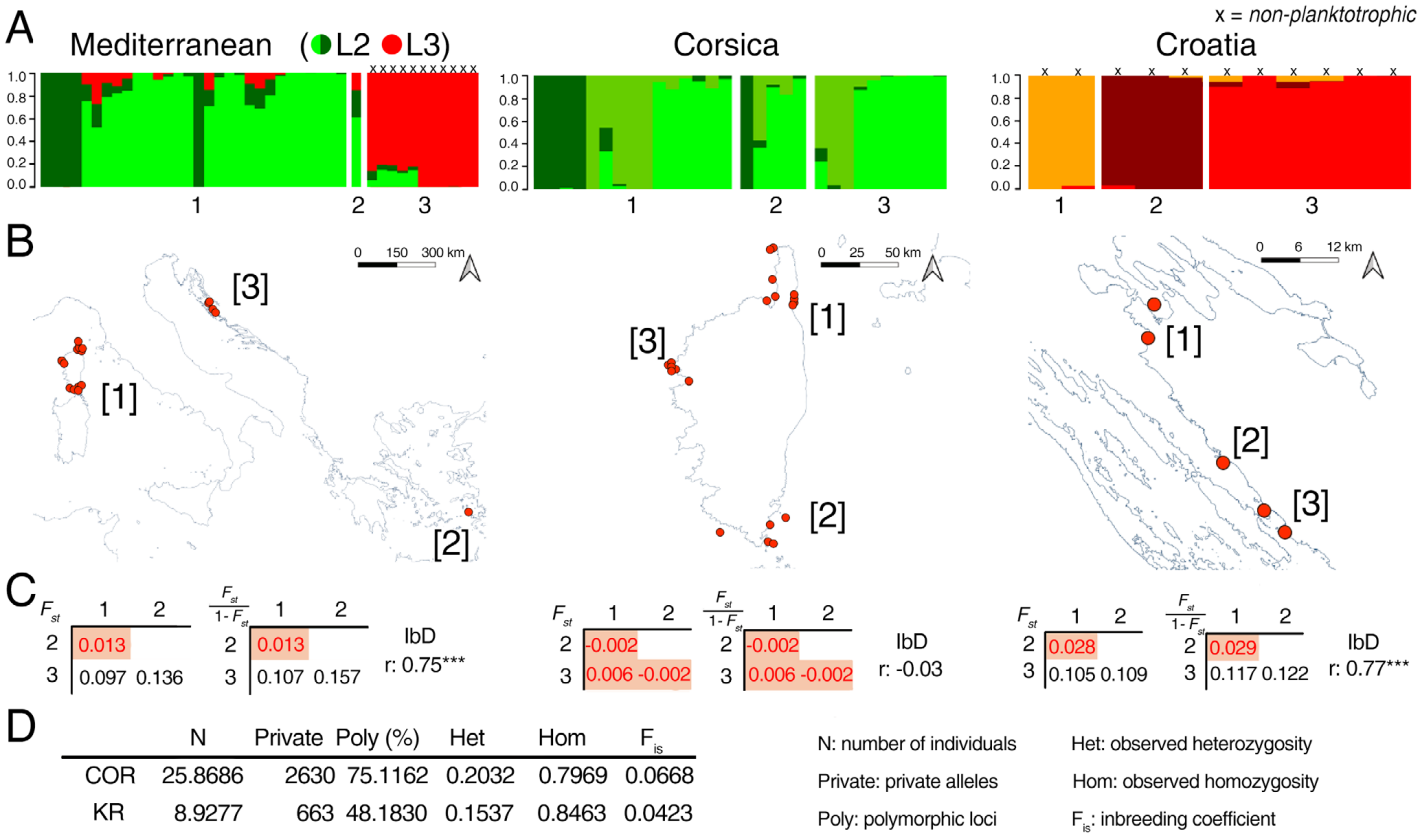
(A) Results of LEA population ancestry analysis of *R. laviae* (L2 + L3) based on geographic localities. Specimens with non‐planktotrophic development marked with ‘*x*’. (B) Sampling map. (C) *F*_*st*_ statistics, with values below the 0.05 threshold highlighted in red and Mantel test correlation index with asterisks representing statistical significance (one: *P‐val* < 0.01, two: *P‐val* < 0.001, three: *P‐val* < 0.0001). (D) Population genetic statistics (COR: Corsica; KR: Croatia).

The patterns resulting from the SNP dataset analyses did not completely match those retrieved in the past from mitochondrial and nuclear markers. However, at least one unit (L2 + L3) comprising enough planktotrophic and non‐planktotrophic specimens was identified. We chose this subsample as our study unit to analyse the effect of poecilogony on population genetics (Figure 4, additional plots in Figure S4). The samples spanned all areas of sampling, with 30 specimens from Corsica (planktotrophic), 1 from Greece (planktotrophic) and 11 from Croatia (non‐planktotrophic).

*F*_*st*_ values were higher than 0.05 between Croatia and the other regions, and also within Croatia between localities 15 and 40 km apart. *F*_*st*_ values between Corsica and Greece and within Corsica were < 0.02, suggesting a single metapopulation. Observed heterozygosity in Corsica (0.20 ± 0.05) and Croatia (0.15 ± 0.11) was congruent with the expected values. The population in Croatia had a lower percentage of private alleles (663 v. 2630) and polymorphic loci (48% v. 75%). The inbreeding coefficient (*F*_*is*_) was also lower (0.04 v. 0.07), but fell within the same confidence interval. The populations of this poecilogonic species retrieved from the structure analysis partially corresponded to those preliminarily assumed on a geographic basis. The non‐planktotrophic samples from Croatia all shared the same ancestral population and Greece was not set apart from Corsica, where two different haplotypes were retrieved. Within Corsica, no geographic pattern was present and Isolation by Distance was not retrieved. On the contrary, a clear structure emerged within Croatia, with a significant (*p* < 0.0001) correlation index of 0.77.

## 4 Discussion

### 4.1 Poecilogony in Raphitoma

Compared to the previous molecular assessments of this group (Chiappa et al. 2025; Russini et al. 2020), our large‐scale genotyping yielded a molecular framework with a much higher resolution, retrieving differences among samples that did not emerge from the previous dataset of few Sanger genetic markers. With only a few exceptions in *R. philberti* (which were resolved when using a set of loci correlated with differences in development), samples of different larval development were segregated, and not mixed as appeared using predominantly mitochondrial markers. Nevertheless, the *gdi* and genetic distance analyses suggested that the distance retrieved is not enough to confidently separate them as distinct species, as occurred with other evaluations of poecilogony cases in shelled gastropods (Fassio et al. 2022). In fact, all *gdi* values between the two units of *R. laviae* with different development L2 and L3 were well below the threshold for conspecificity (Jackson et al. 2017), and the same result was verified from nucleotide diversity estimates, following Roux et al. (2016). Therefore, we were able to assume the specimens in the L2 and L3 groups as comprising a single poecilogonic species, which would result in a typical case of geographic poecilogony. One group, ranging from Corsica to Greece, shows the metapopulational features of planktotrophic developers: it has a wide range and no geographic structure. Although different haplotypes are retrieved in Corsica (Figure 4), these specimens may have originated from very far regions of the Mediterranean (e.g., Eastern v. Western, Alboran Sea v. Central Mediterranean). The population in Croatia, on the contrary, shows a distinctive geographic structure, even at a small geographic scale, which fits an intracapsular development with very limited dispersal abilities.

In Croatia, the lack of recruitment of planktotrophic specimens of this species may indicate that planktotrophic specimens from outside Croatia cannot reach this region anymore. The isolation of this restricted region may be the reason that promoted the loss of planktotrophy in this population, which combined with the prolonged absence of gene flow from outside may eventually prompt a speciation event (Krug 2011; Oliverio 1996). In this case, the barrier to gene flow between the planktotrophic and non‐planktotrophic units in *R. laviae* may be merely of a geographic nature. An alternative explanation is that a drastically lower proportion of planktotrophic specimens can be found in the area at a given time, as their offspring would rapidly switch to a non‐planktotrophic development. This would explain why they are not easily found sympatrically; however, in this case, one would expect not all non‐planktotrophic specimens to be strictly related, since some of them should have originated from planktotrophic ancestors carrying haplotypes of distant populations. This did not emerge from our assessment, but we cannot completely exclude this hypothesis given the sample size of our Croatian dataset, and the absence of sampling from intermediate regions between those where our planktotrophic and non‐planktotrophic populations reside. If there is a transition from planktotrophic to non‐planktotrophic development in the Adriatic (and/or the eastern Mediterranean: Oliverio 1996) favoured by the environmental conditions, with traces of gene flow from the planktotrophic towards the non‐planktotrophic population, it may result from additional sampling in the Southern Adriatic, the Aegean Sea and the Levant basin.

In either case, if loss of planktotrophy were an irreversible process, strong enough to determine by itself a reproductive barrier between samples with different larval development (e.g., by involving asynchronous reproductive events), then poecilogony in those gastropods would be a very transient phenomenon (thus explaining its rarity), with the rapid emergence of reproductively isolated lineages and the following possible destinies: (a) both lineages persist as distinct species, (b) the non‐planktotrophic lineage becomes extinct, as it is more vulnerable to changes in environmental conditions and should therefore be naturally more prone to extinction (especially if limited to a restricted range) and (c) the planktotrophic lineage becomes extinct due to counter‐selection over a wide range, and is replaced by the non‐planktotrophic one. At any short time frame, the perception of the researcher would be that poecilogony does not exist (Bouchet 1989).

Possible drivers of a switch to the non‐planktotrophic strategy could also be related to trophic resources for planktotrophic larvae being insufficient or unpredictable (Krug 2009; Oyarzun and Strathmann 2011). Additionally, or alternatively, sea level lowering may have caused significant changes in hydrographical conditions, producing changes in circulation patterns and confinement of subbasins. Noteworthy, the estimated divergence time of the two species in the pair *R. cordieri–R*. *horrida* (~3.3 mya) correlates with the Late Pliocene Marine Isotope Stage M2, a globally recognisable cooling event, which has disturbed an otherwise relatively warm background climate state, and may have involved a global sea‐level fall of between 40 m and 60 m (Dolan et al. 2015). Similarly, the differentiation within the *R. laviae–R. philberti* complex has started in the Early Pleistocene, i.e., the period following the onset of the glaciations and their southward extension (Thunnel and Douglas 1983, 1984). There is evidence that during glacial periods the Mediterranean underwent severe changes in the circulation patterns, often showing repeated strong confinement of the subsidiary basins (Med v. Atlantic, eastern v. western Med) (Guo et al. 2020; Kaboth et al. 2017), enhanced by sea‐level lowering that caused reductions of the Sicily Channel up to three quarter width (Bethoux 1979, 1984). Under such conditions, fluctuations in the energy (food) input, restricted areas and higher predatory pressure may have easily been the main factors counterselecting the planktotrophic larvae (Strathmann 1978b, 1978a, 1985) in the Eastern Mediterranean.

Finally, the pattern emerged that *R. philberti* had many similarities to *R. laviae*, as non‐planktotrophic specimens were mostly spread according to sampling locality, while the planktotrophic ones were not. Most non‐planktotrophic specimens were also from Croatia, suggesting similar conditions promoting loss of planktotrophy in the genus *Raphitoma*, but two of them were from the Tyrrhenian Sea. Therefore, in this species, we observed the sympatric occurrence of specimens with different larval development, and we also retrieved one non‐planktotrophic sample genetically mixed with planktotrophic ones. Furthermore, when we used the same loci that in *R. laviae* correlated to different larval development, we observed a clear segregation of samples also in *R. philberti*. This result suggests not only that the switch to a non‐planktotrophic strategy may have a genetic component, but also that it could involve the same genes in both species. Unfortunately, the reduced sampling size for this taxon prevented us from analysing in depth its population genetics and drawing evolutionary conclusions on how it is affected by poecilogony.

### 4.2 Alternative Scenarios

Despite our focus being on the validation and study of poecilogony, our analyses also highlighted persisting uncertainties in the current species delimitation of the group. Our starting hypothesis of delimitation was based primarily on *cox‐1* mitochondrial data, which is a robust marker for such aims (Miralles et al. 2024) with wide applications in gastropods (Fedosov and Puillandre 2012; Furfaro et al. 2023; Nocella et al. 2024), although in the most recent analyses (Chiappa et al. 2025) data did not show a clear barcoding gap in this group. Even using hundreds of loci, part of the species delimitation of this group remains in a grey zone (Jackson et al. 2017; Roux et al. 2016). The high co‐ancestry values between *R. philberti* and *Raphitoma* sp. C, their distribution in the PCA and structure analyses and their relatively low genetic divergence, do not unequivocally support their separation as distinct species or their pooling into a single one. Conversely, *R. laviae* as now conceived could comprise multiple taxa (e.g., one planktotrophic species [L1] with wide distribution and one poecilogonic species [L2 + L3], which is the scenario explored in this study).

Evolutionary hypotheses on a group with such quickly changing taxonomy are inevitably burdened by a certain degree of uncertainty (Pante et al. 2015). This is even worse when attempting to validate poecilogony, as the species boundaries between and among planktotrophs and non‐planktotrophs cannot easily be defined without performing cross‐fertilisation experiments. This study explores a likely scenario, assuming in *R. laviae* two different species, one planktotrophic with a wide distribution and one poecilogonic, with different larval development in the two geographic localities explored. However, the taxonomy of this complex is yet to be fully resolved, which should be the aim of a species delimitation study done in an integrative approach. For example, another possible interpretation of the data is that all specimens of *R. laviae* included in the analysis were conspecific, including those in group L1. In this scenario, there is a single metapopulation in the Mediterranean, due to the high dispersal of planktotrophic specimens that may have maintained important gene flow over large distances, maintaining a condition of panmixia. Yet, the strong genetic divergence among groups would indicate a sort of isolation between the sympatric (often syntopic) subpopulations. This would be compatible with the high‐dispersal capability of planktotrophic specimens. In fact, although being sampled together, these specimens may have originated in different, distant regions in the Eastern, Western and Central Mediterranean. Finally, in this scenario, sympatric specimens of differing larval morphology coexist in Croatia. Population genetic statistics for this scenario were computed as they may provide the basis for compelling interpretations of the origin and effect of poecilogony in future studies, and are shown in Figures S5 and S6. Another possibility is that L2 and L3 constitute two different species, and there is no poecilogony in *R. laviae*. The low *gdi* and distance estimates may be explained by a recent speciation event. In this scenario, an allopatric speciation event occurred in the Adriatic Sea, with loss of planktotrophy in the new lineage.

In order to face these unsolved issues, as well as further pursue the study of the genetics underlying poecilogony, it will be necessary to take a step further and analyse the genomes of specimens of this complex. This would allow for the performance of association studies (Uffelmann et al. 2021) and draw evolutionary scenarios that better explain the reasons behind the contrasting levels of polymorphism detected by mitochondrial and nuclear data (Singhal et al. 2025). A second promising strategy to study poecilogony could be a comparative transcriptomic approach applied to eggs/embryos (Lesoway et al. 2016), and to the female gonads, to explore if and how the mother effect is implied in the offspring larval development.

## Author Contributions

Conceptualisation: G.C.; G.F.; M.O.; Data curation: G.C.; N.P.; Formal analysis: G.C.; Investigation: G.C.; G.F.; M.O.; Methodology: G.C.; G.F.; M.V.M.; M.O.; Software: G.C.; Visualisation: G.C.; G.F.; M.O.; Writing original draft: G.C.; Writing – review and editing: G.C.; G.F.; M.V.M.; N.P.; M.O.; Supervision: G.F.; M.V.M.; M.O.; Validation: G.C.; G.F.; M.O.; Resources: G.F.; N.P.; M.O.; Funding acquisition: G.C.; G.F.; N.P.; M.O.; Project administration: M.O.

## Disclosure

Benefit‐sharing: The material was collected during several expeditions operated under the regulations then in force in the countries in question and satisfies the conditions set by the Nagoya Protocol for access to genetic resources. All relevant data and results produced in this study have been shared on appropriate public databases as described above.

## Conflicts of Interest

The authors declare no conflicts of interest.

## Supporting information


**Data S1:** mec70170‐sup‐0001‐Supinfo1.docx.

## Data Availability

Raw sequence reads have been deposited in the SRA repository (www.ncbi.nlm.nih.gov/sra) with BioProject ID PRJNA1242061. The Biosample accession numbers are reported in Table 1. The unfiltered variant call file (vcf) was uploaded to Zenodo with the following doi: 10.5281/zenodo.17086275.
