# Supplementary material for: What Is the Population Structure of Poecilogonic Species? Evidence From Large‐Scale Genotyping in a Neogastropod Lineage (Conoidea: Raphitoma)
Source: Mol Ecol. 2025 Nov 11;34(24):e70170. doi: 10.1111/mec.70170 (PMC12717971; doi:10.1111/mec.70170)
Supplement: Supplementary file 1 — Data S1: mec70170‐sup‐0001‐Supinfo1.docx. [file MEC-34-e70170-s001.docx]

**Supplemental Information for:**

**What is the population structure of poecilogonic species? Evidence from large scale genotyping in a neogastropod lineage (Conoidea: *Raphitoma*)**

Giacomo Chiappa^1*^, Giulia Fassio^1^, Maria Vittoria Modica^2^, Nicolas Puillandre^3^, & Marco Oliverio^1^

^1^Department of Biology and Biotechnologies “Charles Darwin”, Sapienza University of Rome, Viale dell’Università 32, 00185 Rome, Italy; [giacomo.chiappa@uniroma1.it](mailto:giacomo.chiappa@uniroma1.it), [giulia.fassio@uniroma1.it](mailto:giulia.fassio@uniroma1.it), [marco.oliverio@uniroma1.it](mailto:marco.oliverio@uniroma1.it).

^2^Department of Biology and Evolution of Marine Organisms, Stazione Zoologica Anton Dohrn, Via Gregorio Allegri 1, 00198 Rome, Italy; [mariavittoria.modica@szn.it](mailto:mariavittoria.modica@szn.it).

^3^Institut Systématique Evolution Biodiversité (ISYEB), Muséum National d’Histoire Naturelle, CNRS, Sorbonne Université, EPHE, Université des Antilles, 57 rue Cuvier, CP 51, 75005 Paris, France; [puillandre@mnhn.fr](mailto:puillandre@mnhn.fr).

**Table of Contents:**

| **Appendix S1.** Bpp and time estimation analyses calibration | Page 2 |
| --- | --- |
| **Appendix S2.** Supplementary bibliography | Page 3 |
| **Figure S1.** Calibrated tree of Raphitomidae | Page 4 |
| **Figure S2.** Structure analysis (K1–10) plots | Page 5 |
| **Figure S3.** PCA plots of fifth to eighth PCs | Page 6 |
| **Figure S4.** Structure analysis of *Raphitoma laviae* (L2+L3) | Page 7 |
| **Figure S5. S**tructure analysis of *Raphitoma laviae* (L1+L2+L3) | Page 8 |
| **Figure S6.** Population structure by geographic localities  of *Raphitoma laviae* (L1+L2+L3) | Page 9 |

**Appendix S1-1.** Bpp analysis calibration.

The SLAF tags datasets were analysed with a Bayesian approach using the species delimitation algorithm bpp 4.0 (Flouri et al., 2018; Yang, 2015). We used the genealogical divergence index (*gdi*) from Jackson et al. (2017) to evaluate whether the clades identified in the structure analyses were conspecific (Poelstra et al., 2021; Rennala & Yang, 2020).

The root age (*τ*) and genetic diversity (*θ*) priors were estimated for each subset using the A00 bpp algorithm with three starting sets of divergence time parameters: *τ* = 0.005*θ*, *τ* = 0.05*θ,* and *τ* = 0.5*θ*, with *θ* = 0.005 following Leaché et al. (2019). For each starting set, new *τ* and *θ* were repeatedly estimated while progressively lumping the groups retrieved in the SNP dataset structure analyses. Each analysis was run for 500 000 MCMC generations with a 50 000 burnin twice (Flouri et al., 2018). Convergence between the two runs was checked with Tracer (Rambaut et al., 2018).

The root age of each pair of groups (*τ_AB_*) was then used with the two genetic diversity priors (*θ_A_* and *θ_B_*) separately to measure the *gdi* following the formula *gdi* = 1 − e^−2^*^τ^*^/^*^θ^*, and consistency was checked among the estimates of each pair, for each dataset.

**Appendix S1-2.** Time estimation analysis calibration.

The optimal smoothing parameters for the penalized likelihood analyses were determined using cross validation tests (Sanderson, 2002), and mrca ages were estimated using the truncated Newton algorithm. Node ages of the Raphitomidae ML tree with 95% confidence intervals were calculated in r8s by analysing 1000 ML bootstrap trees with identical topology and bootstrapped branch length. Bootstrap replicates of the dataset were computed with Seqboot in PHYLYP (Felsenstein, 2009), and ML trees with constrained topology were estimated with PAUP* v. 4.0 (Swofford, 2002). Likelihood-ratio tests (Huelsenbeck & Rannala, 1997) were performed using Modeltest v. 3.7 (Posada & Crandall, 1998) in ‘LRT calculator mode’ and r8s v 1.71 (Sanderson, 2003), to test a constant evolutionary rate among different lineages across the tree (i.e. existence of a molecular clock), and whether the two lineages descending from each node evolved at the same constant rate.

**Appendix S2.** Supplementary bibliography.

Bianchini, G., & Sánchez‐Baracaldo, P. (2024). TreeViewer: Flexible, modular software to visualise and manipulate phylogenetic trees. *Ecology and Evolution*, *14*(2), e10873. https://doi.org/10.1002/ece3.10873

Flouri, T., Jiao, X., Rannala, B., & Yang, Z. (2018). Species tree inference with BPP using genomic sequences and the Multispecies Coalescent. *Molecular Biology and Evolution*, *35*(10), 2585–2593. https://doi.org/10.1093/molbev/msy147

Frichot, E., & François, O. (2015). LEA: An R package for landscape and ecological association studies. *Methods in Ecology and Evolution*, *6*(8), 925–929. https://doi.org/10.1111/2041-210X.12382

Jackson, N. D., Carstens, B. C., Morales, A. E., & O’Meara, B. C. (2017). Species delimitation with gene flow. *Systematic Biology*, *66*(5), 799–812. https://doi.org/10.1093/sysbio/syw117

Leaché, A. D., Zhu, T., Rannala, B., & Yang, Z. (2019). The spectre of too many species. *Systematic Biology*, *68*(1), 168–181. https://doi.org/10.1093/sysbio/syy051

Poelstra, J. W., Salmona, J., Tiley, G. P., Schüßler, D., Blanco, M. B., Andriambeloson, J. B., Bouchez, O., Campbell, C. R., Etter, P. D., Hohenlohe, P. A., Hunnicutt, K. E., Iribar, A., Johnson, E. A., Kappeler, P. M., Larsen, P. A., Manzi, S., Ralison, J. M., Randrianambinina, B., Rasoloarison, R. M., … Yoder, A. D. (2021). Cryptic Patterns of Speciation in Cryptic Primates: Microendemic Mouse Lemurs and the Multispecies Coalescent. *Systematic Biology*, *70*(2), 203–218. https://doi.org/10.1093/sysbio/syaa053

Rambaut, A., Drummond, A. J., Xie, D., Baele, G., & Suchard, M. A. (2018). Posterior Summarization in Bayesian Phylogenetics Using Tracer 1.7. *Systematic Biology*, *67*(5), 901–904. https://doi.org/10.1093/sysbio/syy032

Rennala, B., & Yang, Z. (2020). Species delimitation. In *Phylogenetics in the Genomic Era* (pp. 1–18). Celine Scornavacca, Frédéric Delsuc and Nicolas Galtier; https://hal.inria.fr/PGE

Yang, Z. (2015). The BPP program for species tree estimation and species delimitation. *Current Zoology*, *61*(5), 854–865. https://doi.org/10.1093/czoolo/61.5.854


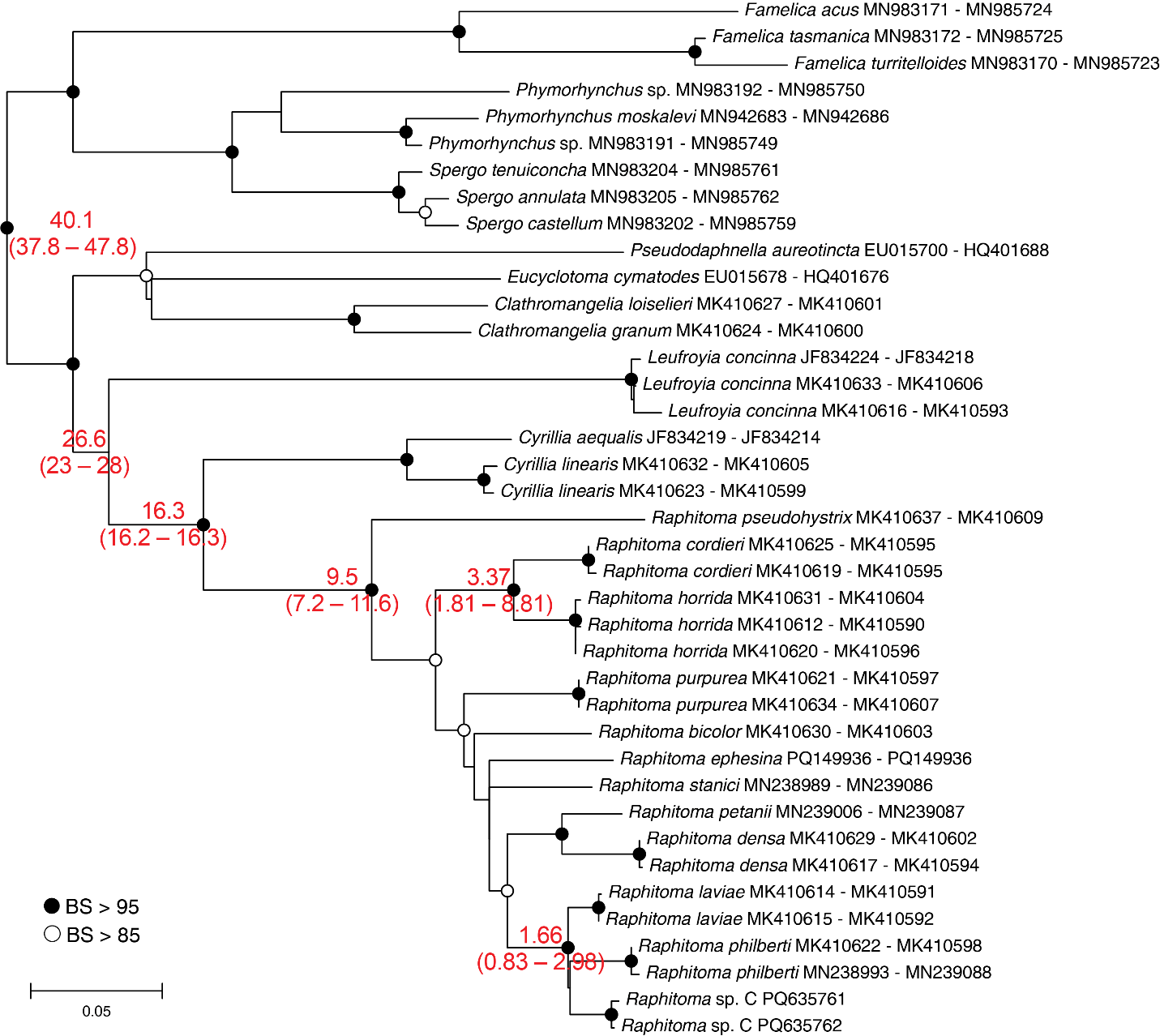


**Figure S1.** Maximum likelihood phylogenetic reconstruction of Raphitomidae, with estimated node ages (mya) in red. Circles at nodes indicate 1000 ultrafast bootstrap values. Tree edited in TreeViewer v2.2.0 (Bianchini & Sánchez‐Baracaldo, 2024).


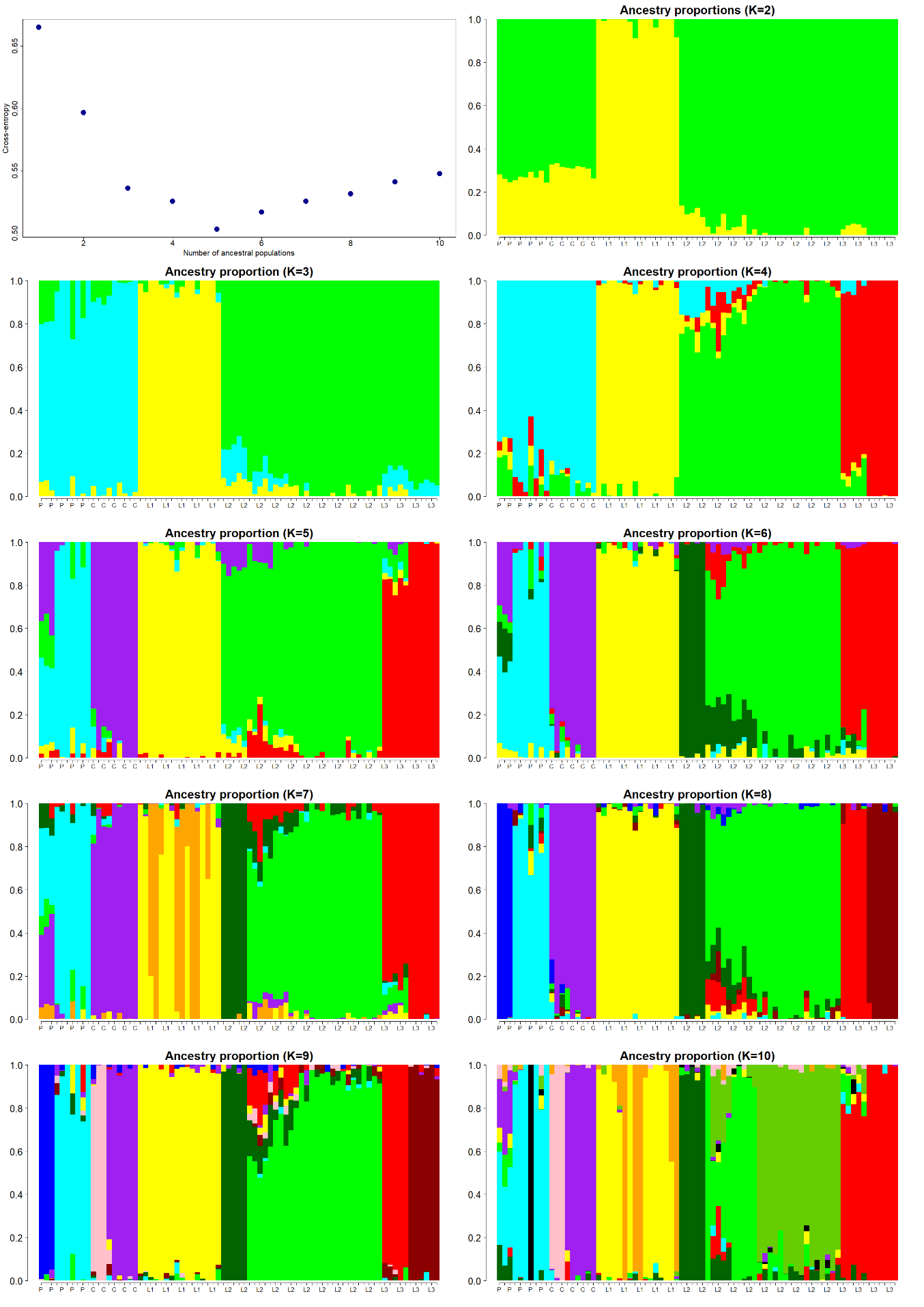


**Figure S2.** Cross-entropy plot and shared ancestry of the LEA package structure analysis (Frichot & François, 2015), obtained using a dataset of 1789 loci (one random SNP per locus).


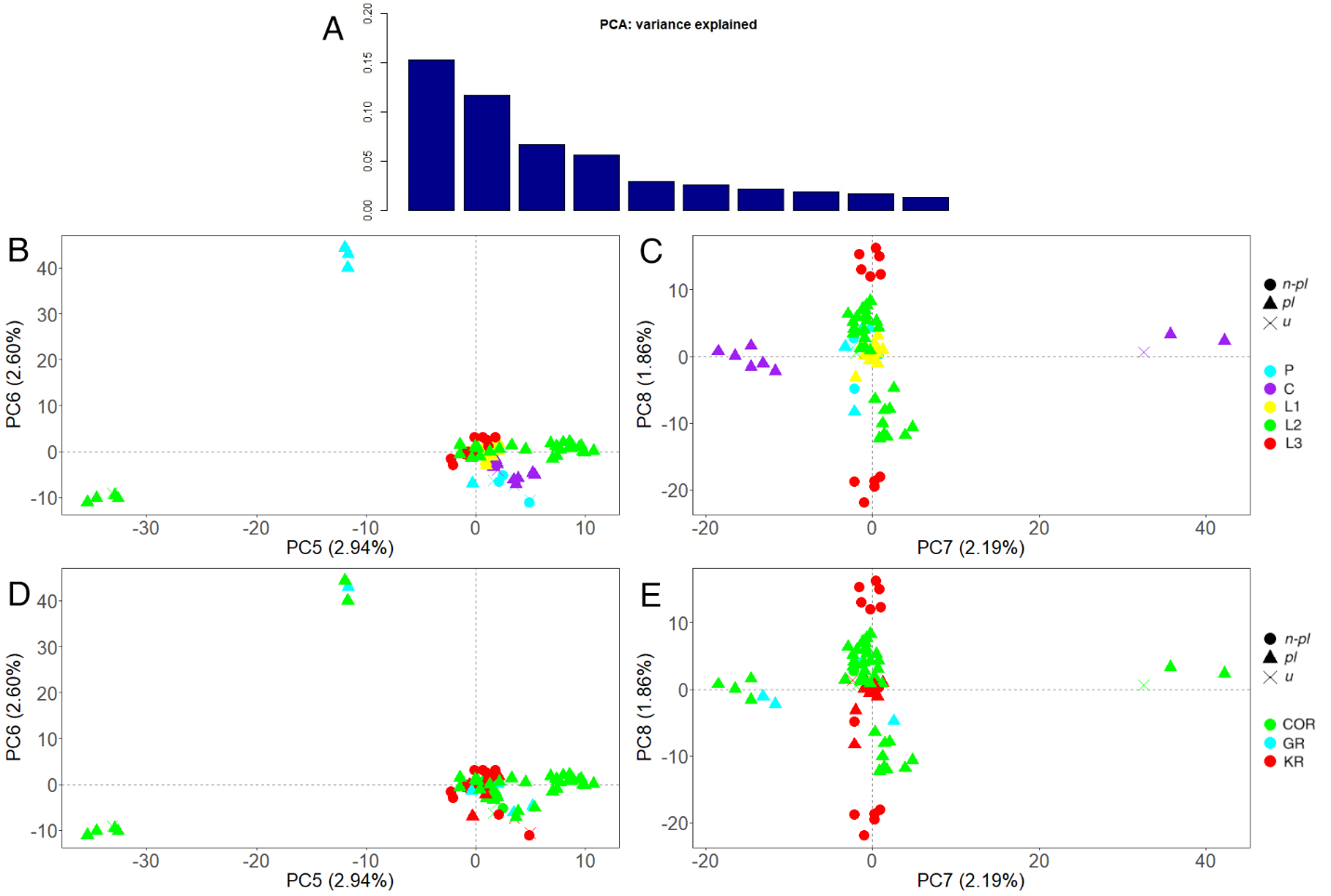


**Figure S3.** PCA plots with the fifth to eighth principal components. **A:** Proportion of variance explained by the first ten components. **B, C**: Colour legend corresponding to populations retrieved in the structure analyses (Figure 1). **D, E**: Colour legend corresponding to sampling localities (COR: Corsica; GR: Greece; KR: Croatia). Larval development inferred from protoconch morphology (*pl*: planktotrophic; *non-pl*: non-planktotrophic; *u*: unknown).


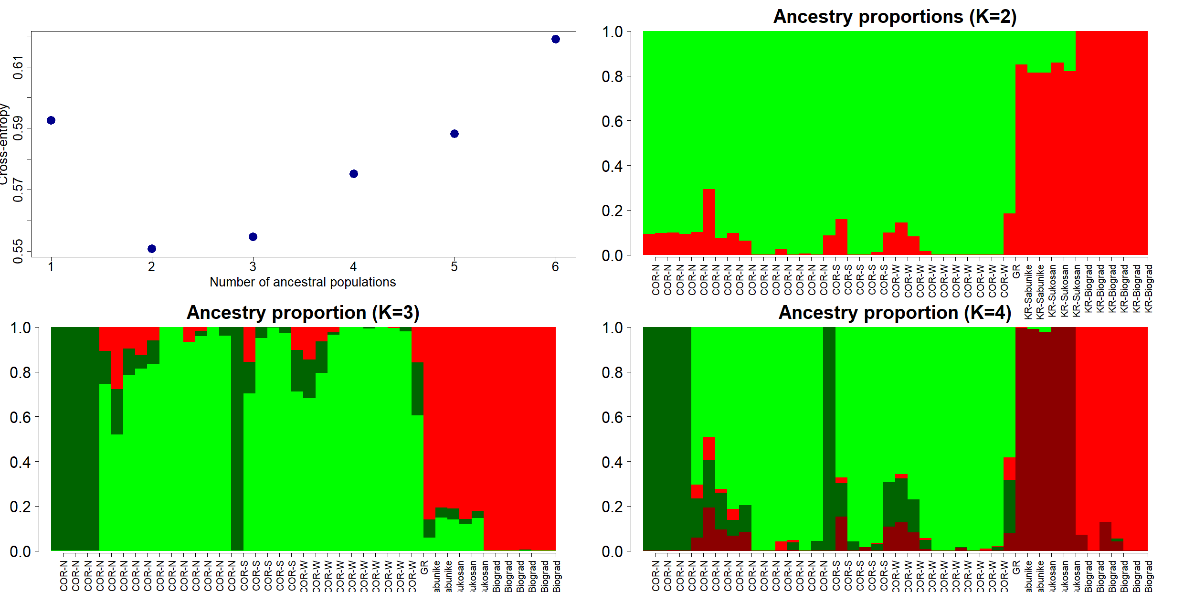


**Figure S4-1.** Cross-entropy and structure plots of the *R. laviae* dataset (L2+L3).


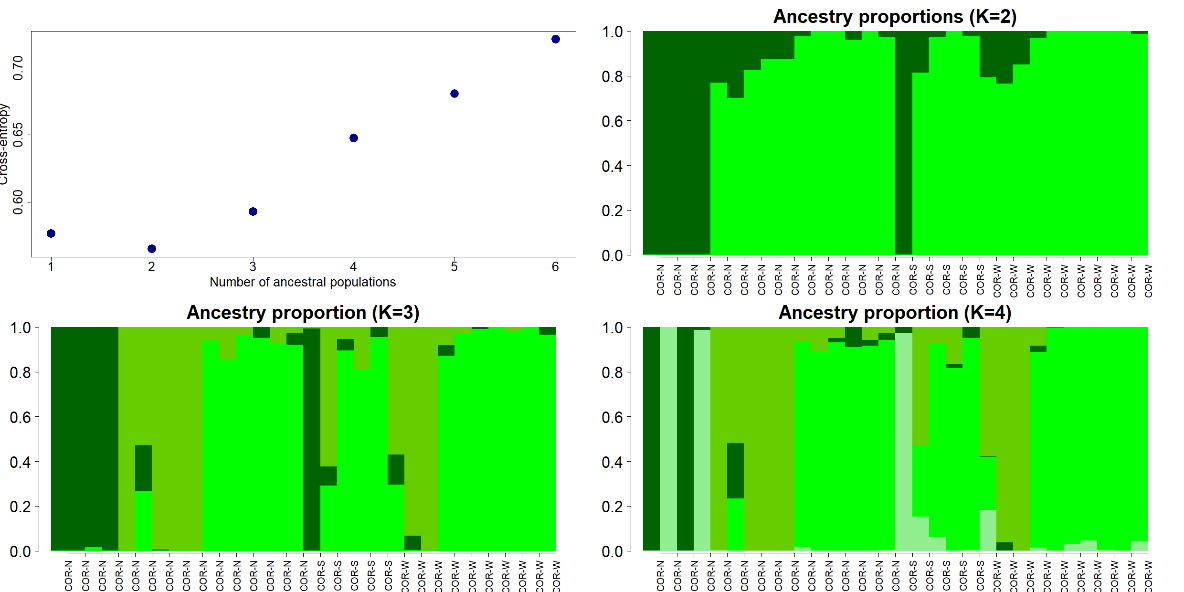


**Figure S4-2.** Cross-entropy and structure plots of the *R. laviae* dataset (L2+L3) in Corsica.


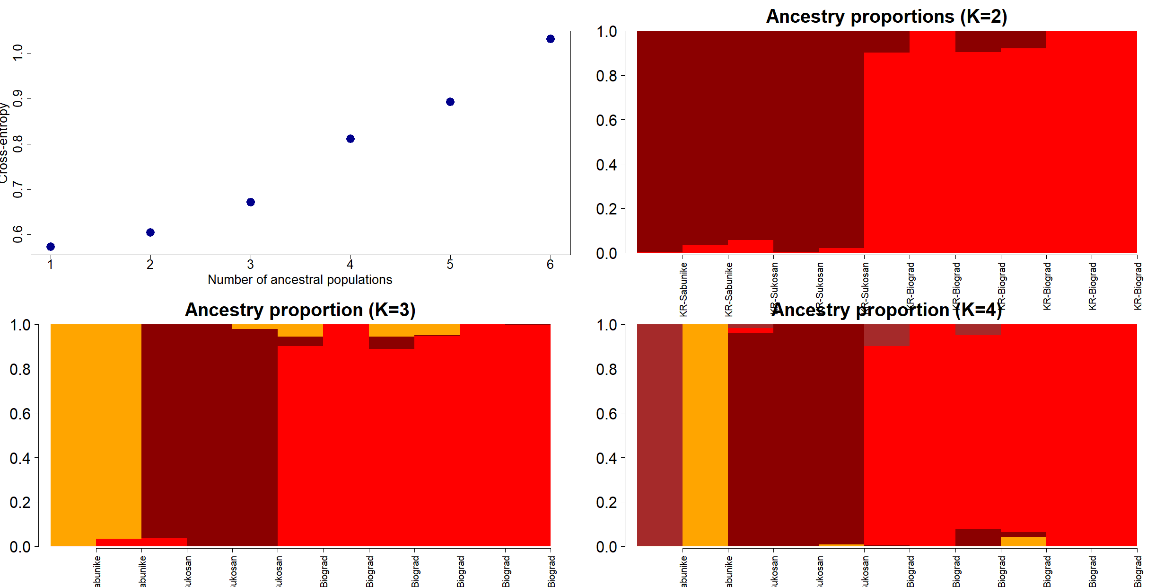


**Figure S4-3.** Cross-entropy and structure plots of the *R. laviae* dataset (L2+L3) in Croatia.


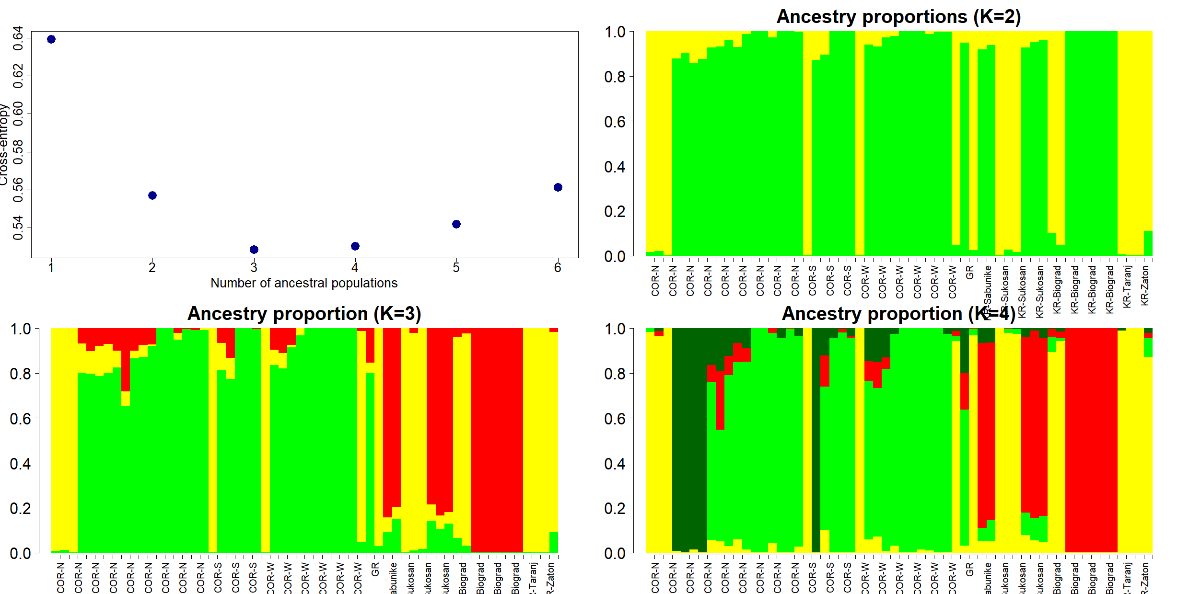


**Figure S5-1.** Cross-entropy and structure plots of the *R. laviae* dataset (L1+L2+L3).


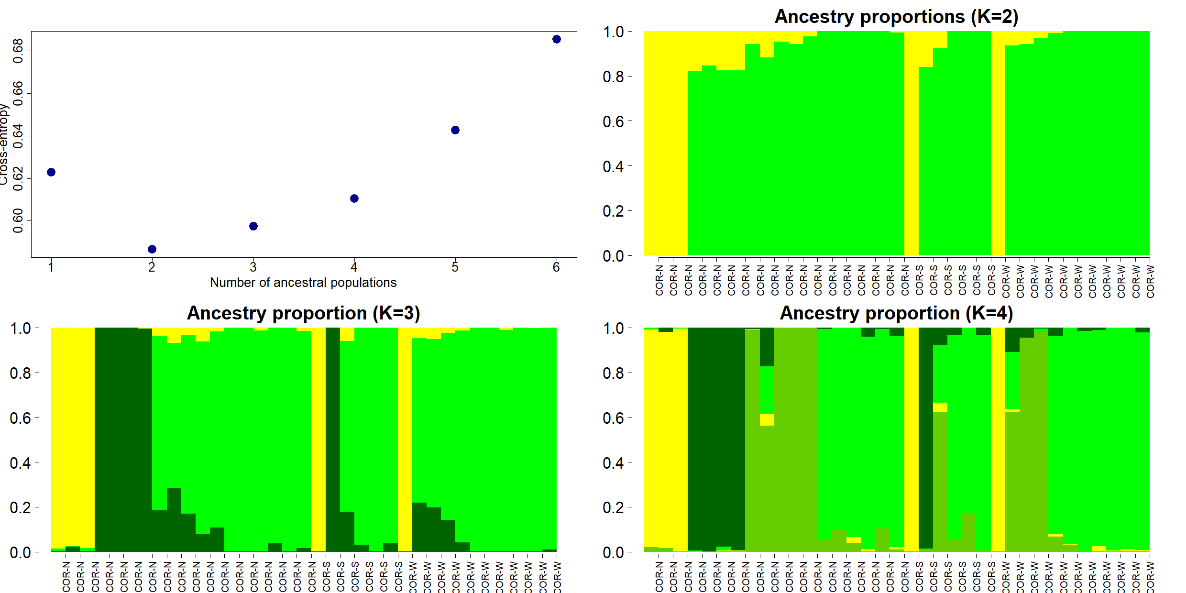


**Figure S5-2.** Cross-entropy and structure plots of the *R. laviae* dataset (L1+L2+L3) in Corsica.


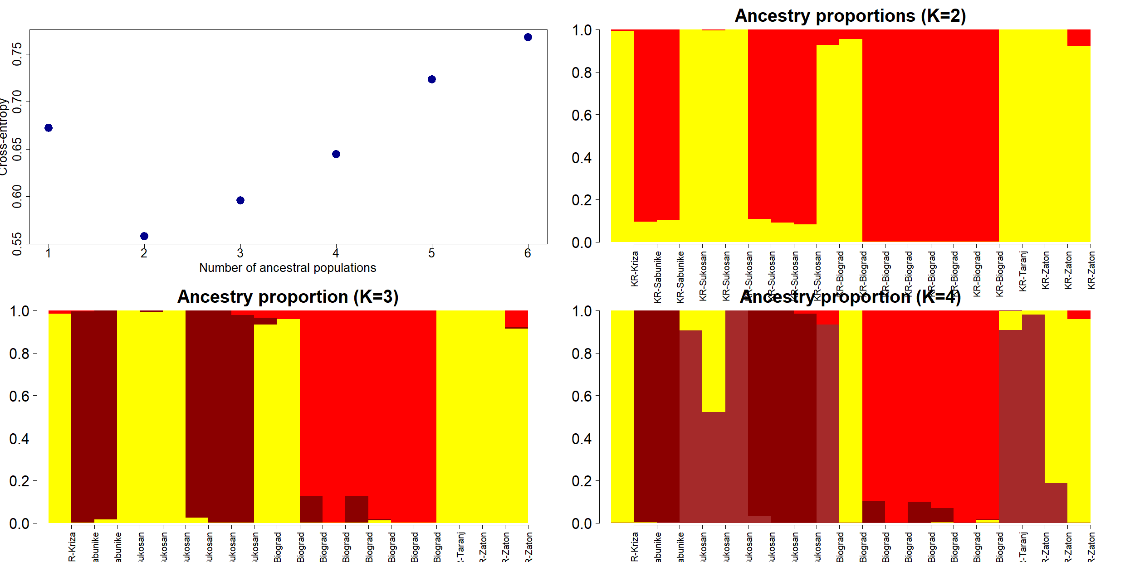


**Figure S5-3.** Cross-entropy and structure plots of the *R. laviae* dataset (L1+L2+L3) in Croatia.


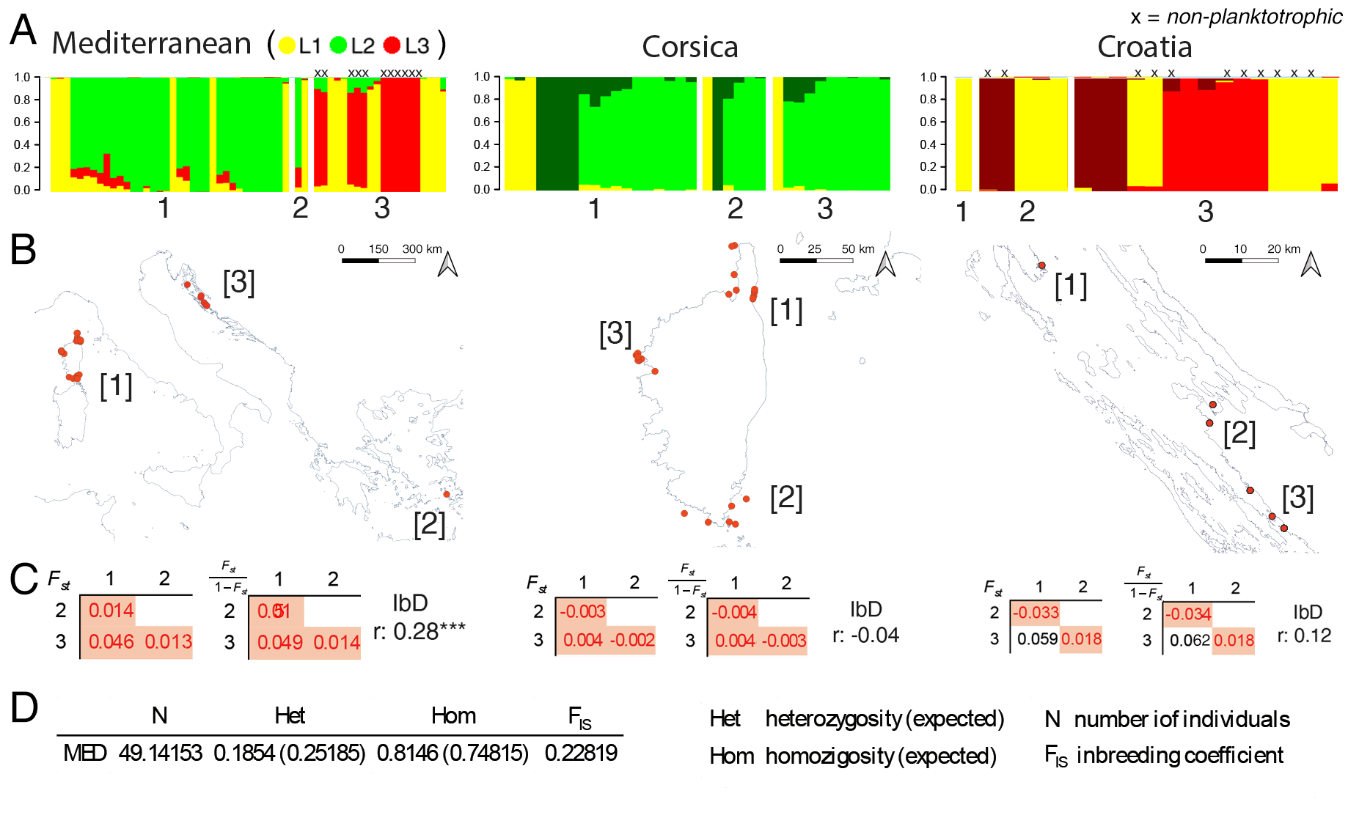


**Figure S6. A.** Results of LEA population ancestry analysis of *R. laviae* (L1+L2+L3) based on geographic localities. Specimens with non-planktotrophic development marked with ‘x’. **B.** Sampling map. **C.** F_st_ statistics, with values below the 0.05 threshold highlighted in red and Mantel test correlation index with asterisks representing statistical significance (one: *p-val* < 0.01, two: *p-val* < 0.001, three: *p-val* < 0.0001). **D.** Population genetic statistics (COR: Corsica; KR: Croatia).
